# Optimizing Deep Learning-Based Segmentation of Densely Packed Cells using Cell Surface Markers

**DOI:** 10.21203/rs.3.rs-3307496/v1

**Published:** 2023-09-26

**Authors:** Sunwoo Han, Khamsone Phasouk, Jia Zhu, Youyi Fong

**Affiliations:** Vaccine and Infectious Disease Division, Fred Hutchinson Cancer Research Center, Seattle, USA

**Keywords:** computer vision, deep learning, cell segmentation, HSV

## Abstract

**Background:**

Spatial molecular profiling depends on accurate cell segmentation. Identification and quantitation of individual cells in dense tissues, e.g. highly inflamed tissue caused by viral infection or immune reaction, remains a challenge.

**Methods:**

We first assess the performance of 18 deep learning-based cell segmentation models, either pre-trained or trained by us using two public image sets, on a set of immunofluorescence images stained with immune cell surface markers in skin tissue obtained during human herpes simplex virus (HSV) infection. We then further train eight of these models using up to 10,000+ training instances from the current image set. Finally, we seek to improve performance by tuning parameters of the most successful method from the previous step.

**Results:**

The best model before fine-tuning achieves a mean Average Precision (mAP) of 0.516. Prediction performance improves substantially after training. The best model is the cyto model from Cellpose. After training, it achieves an mAP of 0.694; with further parameter tuning, the mAP reaches 0.711.

**Conclusion:**

Selecting the best model among the existing approaches and further training the model with images of interest produce the most gain in prediction performance. The performance of the resulting model compares favorably to human performance. The imperfection of the final model performance can be attributed to the moderate signal-to-noise ratio i the imageset.

## Introduction

**Figure 1:**
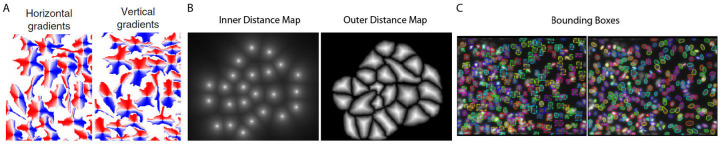
All methods model whether a pixel is inside or outside a cell. In addition, (A) Cellpose models gradient flow derived through simulated diffusion ([9] Fig 1c); (B) DeepDistance and DeepCell model inner and outer distance maps derived from cell centers and boundaries ([13] Fig 2b and 2c); (C) Mask R-CNN models bounding boxes, which are rectangular regions surrounding each cell ([21] Fig 16b and 16c).

A major task in imaging analysis is to accurately identify (or segment) individual cells within an image. While manual cell segmentation has often been conducted because it does not require extra computational tools and utilizes human expertise, it is not scalable and has low reproducibility. As an alternative, traditional automated cell segmentation has been researched for many years (e.g. [1] and references within). Moreover, recent advances in computer vision have spurred a new generation of state-of-the-art deep learning-based cell segmentation models [2–11]. Among these deep learning-based models, three approaches stand out: Cellpose, DeepCell/DeepDistance, and Mask R-CNN (Figure 1). All three try to predict whether a pixel is inside or outside a cell, but an inside-outside map alone is not su cient for cell segmentation. These approaches di er in how they identify individual cells. Cellpose and DeepCell/DeepDistance combine deep learning and traditional cell segmentation methods and are specifically designed for cell segmentation. Cellpose [9, 12] models the horizontal and vertical gradients of a topological map with a single smooth basin, which can be used to infer a cell through gradient tracking. DeepDistance [13] and DeepCell [10] formulate cell detection as a problem of finding cell centers and solve the problem through modeling distances to cell centroids and closest cell boundary. On the other hand, Mask R-CNN [14–18] is a general-purpose instance segmentation method, which models a rectangular bounding box around each object. It has been adapted to cell segmentation in e.g., CellSeg [11]. All three approaches have been demonstrated to be capable of achieving near human expert-level performance for images containing well-separated cells. However, high cell density and limited image resolution are known to impact model performance [11, 19, 20].

Our goal is to assess and optimize the performance of deep learning-based cell segmentation models on a set of immunofluorescence images of herpes simplex virus (HSV)-infected skin lesion. HSV causes recurrent mucocutaneous diseases worldwide. To gain insight into the battlefields of genital herpes infection, multiplexed immunofluorescence-based imaging approaches are used for cellular classification and functional distinction to characterize the local inflammatory responses. However, HSV-infected lesions contain densely infiltrated immune cells that are tightly packed together and vary in cellular size, morphology, and phenotype, making precise cell segmentation challenging. It is an open question whether existing cell segmentation models are capable of achieving expert-level performance on such imagesets.

The rest of the paper is organized as follows. In the results section we first report a new imageset of annotated immunofluorescence images of HSV lesion tissue that has been immunostained for the immune cell surface markers CD3, CD4, and CD8. The imageset contains over 12,000 expert-drawn cell masks, which we divide into a training set and a test set. We then evaluate the performance of 18 Cellpose, DeepCell, and Mask R-CNN models trained using the Cellpose, TissueNet [10], and BBBC038v1 (Kaggle 2018 Data Science Bowl dataset from the Broad Bioimage Benchmark Collection) [22] imagesets. Next, we further train five Cellpose models, two DeepCell models, and one Mask R-CNN model with our imageset and examine how the performance changes with increasing training dataset size. The performance of Cellpose models stands out and we seek to further improve the accuracy of predictions by optimizing various aspects of Cellpose training and inference. Lastly, we visually compare the predicted masks before and after further training. We end the paper with a discussion on the achievements and limitations of the existing cell segmentation models and touch on directions for future research.

## Methods

Immunofluorescence-stained tissue sections of various markers of interest were imaged with a Zeiss Pln Apo 20x/0.8 DICII objective lens with 500 nm/pixel resolution (Canopy Biosciences’ ZellScannerOne imaging system) [23].

To annotate the images, an expert with 10+ years of experience in imaging drew ground truth masks using Fiji’s (ImageJ’s) polygon tracing tool. The masks were drawn on top of images shown at 400% magnification. To provide maximum contrast, the images were shown in grayscale. In addition, various image cues including brightness differences, cell-cell boundaries, and halo effects were utilized to determine cell boundaries, especially in cell clusters. Each cell mask took 15–60 mouse clicks and 20–60 seconds to draw, depending mostly on cell size variability and varying difficulty in deducing cell shape.

Two pre-processing steps were performed before cell segmentation: (1) background reduction and optimizing brightness using the ZKW DataWizard application, and (2) eliminating/blacking out false or artificial fluorescence signals generated by debris introduced into the chip chamber during the multiple staining cycles using Fiji (ImageJ).

All model training and evaluation scripts are contained in the following GitHub repository: https://github.com/youyifong/dense cell Cellpose model training and evaluation were implemented in a series of shell scripts. We used a fork of Cellpose (https://github.com/youyifong/cellpose) to implement changes that allow the modification of some training parameters. DeepCell model training and evaluation were implemented in a series of Jupyter Notebooks. For training with TissueNet 1.0 data, we first extracted the nuclear and cytoplasmic marker training data into separate directories. Each training image was 512×512 pixels. For training with immunofluorescence images of HSV-infected skin lesion biopsies, each training image was rescaled by a factor of 2 and divided into 25 512×512 overlapping patches. CellSeg and JACS model training and evaluation were implemented in a series of python and shell scripts. Please see the readme file in the repository for more details.

The training results for the Cellpose models are numerically reproducible on CPU and GPU. The training results for DeepCell and Mask R-CNN models are numerically reproducible on CPU but not on GPU due to non-determinism in some GPU algorithms.

## Results

### Annotation of immunofluorescence images of inflammatory lesion skin biopsies

We annotated seven images immunofluorescence stained with immune cell surface markers, CD3, CD4 or CD8, and created a total of 12,377 cell masks as ground truth by an expert with 10+ years research experience (Table 1). The images were analyzed in gray scale and at 400% magnification to ensure best quality. Various signal cues, including brightness, cell boundary, and halo effect, were considered in determining individual cells, especially in cell clusters. Assuming that training data enriched in dense regions would help improve model performance the most, we sampled five CD3-stained images, one CD4-stained image, and one CD8-stained image, as all T cells express CD3 and only a subset express CD4 or CD8.

Each image is measured at 1392×1040 pixels. To prevent image-to-image variation from introducing bias into the results, we split each image into a 1159×1040 training portion and a 233×1040 testing portion with a single vertical cut, and did the same on the mask file. After splitting, we identified the masks that fell on the cut line, removed these masks from the mask files, and set the pixel intensities for the area covered by these masks to the background value in the image files. Alternatively, the split can be done by cutting each image into non-overlapping tiles of equal sizes and selecting a random portion for testing. We chose the single cut approach here because there is less information loss due to the cut lines, and non-uniformity of background effects within each image appears minimal in this imageset. The test images were used to examine model performance, while the training images were used to train and improve models in cell segmentation.

### Performance of models trained with Cellpose, DeepCell and BBBC038v1 imagesets

**Figure 2:**
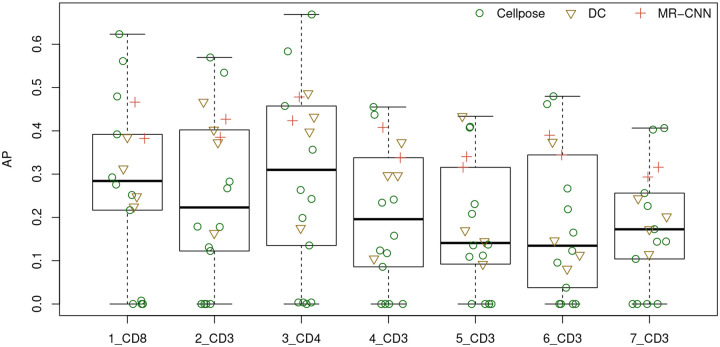
Average precision of the pre-trained models. Each point corresponds to one pre-trained model. DC: DeepCell, MR-CNN: Mask R-CNN.

We compare the prediction performances of 18 models that are either pre-trained or trained by us using TissueNet and BBBC038v1 imagesets (Table 2 and Figure 2). Throughout this paper, we measure prediction performance for a single test image in terms of average precision (AP, also referred to as the Critical Success Index). This metric is calculated as AP = TP/(TP+FN+FP), where true positive (TP) is the number of correctly identified cells, false negative (FN) is the number of actual cells not predicted, and false positive (FP) is the number of predicted cells not corresponding to actual cells. The mean AP value across all test images is referred to as mAP. Since TP, FN, and FP depend on the values used to threshold intersection over union (IoU) between a predicted cell and an actual cell, AP and mAP are also dependent on the threshold. We use a common threshold, 0.5, for the results below.

Cellpose provides a model zoo that contains over a dozen pre-trained models. These models share the same neural network architecture, but differ in their training dataset. The default model is *cyto*, which was trained on about 70,000 cells from six different image types curated by the authors, including fluorescence microscopy images of well-separated neuroblastoma cells from the Cell Image Library [24], brightfield microscopy images, and even non-microscopy images such as apples and jellyfish. A sample neuroblastoma cell image is shown in Supplemental Materials Figure A.1. *Cyto* was shown to perform very well on neuroblastoma cell images, achieving a mAP of around 0.9 [9]. The diversity of the training data also confers a degree of robustness on *cyto* such that it performs reasonably well even on image types on which it was not trained. For example, when applied to the fluorescence- and mass spectrometry-based TissueNet images [10] and the phase contrast images from the LiveCell database [20], cyto has a mAP of around 0.5 and 0.4, respectively [12]. Cellpose also includes *cyto2*, which was trained on the same data as *cyto* plus additional user-contributed images.

Cellpose 2.0 adds two models that are fine-tuned with data from TissueNet and LiveCell. These two models improve the mAP for TissueNet and LiveCell test images to around 0.75 and 0.7, respectively [12]. In addition, due to the heterogeneity of these two large image databases, Cellpose also provides *TN1*, *TN2*, and *TN3*, which are models fine-tuned with distinct subsets of TissueNet, and *LC1, LC2, LC3*, and *LC4*, which are models fine-tuned with distinct subsets of LiveCell.

These 11 Cellpose pre-trained models were trained on a mix of one-channel and two-channel images and can make predictions for either one-channel or two-channel images. The first channel always contains images of cytoplasm or cell surface markers and the optional second channel contains images of nuclear markers. For our images taken from the HSV lesion skin tissues, lymphocyte nuclei DAPI staining is dim and often seen as fused patterns (Supplemental Materials Figure A.3). We thus only used one-channel cell surface marker images in our testing.

Cellpose also includes a pre-trained model *nuclei*, which was trained on one-channel images of nuclei. The types of nuclei images used to train this model are not clear from the online documentation. Although trained on nuclei images, the model can also be used to segment images of cell surface markers because the model is agnostic of the distinction between cells and nuclei. We included this model in our test as well for a total of 12 Cellpose models.

DeepCell provides three pre-trained segmentation models: *CytoplasmSegmentation, NuclearSegmentation*, and *Mesmer*. The first two models share the same model architecture, which introduced a modified inner distance loss function that depends on cell size [10]. During training, the models include an inner distance head, an outer distance head, and a classification head, and expect one-channel image files and one-channel mask files as input. The *CytoplasmSegmentation* model was trained from a computationally curated dataset that comprises pooled phase and fluorescent cytoplasm data, and the *NuclearSegmentation* model was trained from a pooled nuclear dataset from HEK293, HeLa-S3, NIH-3T3, and RAW264.7 cells. As is the case with the Cellpose *nuclei* model, we can use the *NuclearSegmentation* model to segment images of cell surface markers as well.

The last model, *Mesmer*, has a different model architecture from the two previous models. It uses two inner distance heads and two classification heads, and trains on two-channel image files and two-channel mask files, where one channel is for a cytoplasmic or cell surface marker and the other channel is for a nuclear marker. *Mesmer* was trained with TissueNet, which includes over a million cells in total. At prediction, *Mesmer* expects two-channel image files as input and tries to predict every nucleated cell in the image. As the DAPI staining is dim and fused in these tissues and our goal is to predict cells expressing specific markers, we skipped *Mesmer* in our test.

Because the pre-trained *CytoplasmSegmentation* and *NuclearSegmentation* models included in DeepCell are not trainable, to prepare for the next section we implemented a DeepCell model same as the model in *CytoplasmSegmentation* and *NuclearSegmentation* using the DeepCell code. We trained the model with the cytoplasm channels in the image and mask files from TissueNet. There were over 800,000 training instances. A sample image is shown in Supplemental Materials Figure A.2. We followed the training procedure in a training notebook included in DeepCell, and refer to the trained model as DC *tn_cyto*. Similarly, we trained the same model using the nuclear channels in the image and mask files from TissueNet, which included over 700,000 training instances. We refer to this model as DC *tn_nuclear*.

CellSeg provides a single pre-trained model that is based on the Mask R-CNN architecture for general purpose instance segmentation. An important modification to the model it introduced was reducing the contribution of the classification loss in the overall loss function. It was trained with image set BBBC038v1, which contains nearly 30,000 segmented nuclei. CellSeg includes an optional mask expansion step to go from nuclear masks to cell masks. Since we use the model to segment cell surface marker images, we skip the mask expansion step.

Since the pre-trained model provided by CellSeg is not trainable, to prepare for the next section, we implemented a Mask R-CNN model using the Torchvision library and used the CellSeg code to perform post-processing related to stitching together image tiles. We adopted the same modification to the loss function that was introduced by [11], and trained it using image set BBBC038v1. We refer to the model thus derived as Just Another Cell Segmenter (JACS).

All three sets of pre-trained models include parameters that can be adjusted at prediction time. We left all these parameters at default with one important exception - the parameter that controls rescaling of testing images (diameter in Cellpose, image mpp in DeepCell, and INCREASE FACTOR in CellSeg). The default in Cellpose is to estimate the size of cells in the test image in a preliminary run and scale the test image so that the average cell size from the test image matches that of the data used to train the Cellpose model. We chose the default option for all Cellpose models. DeepCell and CellSeg lack such a step to choose rescaling adaptively, so we set the parameter by experimenting with a sequence of values and chose the one with the best mAP. For the two DeepCell models that we trained with TissueNet data, we reuse the rescaling parameter derived for the DeepCell models. For JACS, we found that scaling was effectively controlled by the size of the patches used in evaluation and we chose a patch size that was comparable to the size of the training images.

The APs of all 18 pre-trained models are presented in Table 2 and Figure 2. Based on mAP, the two best models are Cellpose *cyto* (0.516) and *cyto2* (0.484). CellSeg comes in third place with a mAP of 0.404. Among the DeepCell models, *tn_nuclear* (0.387) performs the best and *tn_cyto* (0.140) performs the worst. This is not surprising when we look at a sample image from TissueNet (Figure A.2), which shows the difficulty in defining cell-cell boundaries based on cytoplasmic staining. This may also help explain why the worst performers from Cellpose are *tissuenet* (0.001), *TN1* (0.001), *TN2* (0.001), and *TN3* (0.001), all models that were fine-tuned with TissueNet data (Cellpose training does not use nucleus masks).

Comparing across testing images, Figure 2 shows that the models overall perform better on the CD4 and CD8 images than on the CD3 images, consistent with the fact that cell density is lower in the CD4 and CD8 images than in the CD3 images.

### Training with images of densely packed cells improves prediction

The performance of the pre-trained models showed promise but was not good enough for practical use. We hypothesized that further training of these models with immunofluorescence images of inflammatory lesion skin biopsies could improve prediction accuracy.

All the pre-trained Cellpose models are trainable. We selected four of them for further training: *cyto*, *cyto2*, *tissuetnet*, and *livecell*. *Cyto* and *cyto2* had the best performance among all pre-trained models in the previous section. We included *tissuenet* and *livecell* as a comparison because they were trained with more data than *cyto* and *cyto2*. We also trained a Cellpose model that has random weights for comparison, which we will refer to as none because it was not pre-trained with any data.

The two pre-trained DeepCell models*, CytoplasmSegmentation* and *NuclearSegmentation*, and the Mask R-CNN CellSeg model cannot be fine-tuned because they leave out the binary classification head. However, the two DeepCell models that we trained with TissueNet images, tn cyto and tn nuclear, and the Mask R-CNN model jacs we trained with image set BBBC038v1 can be fine-tuned, and we selected all three of them.

Many parameters can be adjusted in the training stage. The default choices for most of the parameters are reasonable, but one aspect that requires careful consideration is scaling of the new training data to match the data used to pre-train the models. Cellpose handles this issue in a way that requires no user action: all pre-trained cell segmentation models have the same mean cell diameter of 30 and Cellpose estimates the mean cell diameter in the new training images in a preliminary step, as is done for the test images in evaluation. Cellpose then uses the estimated mean cell diameter to compute a scale factor for the new training images. For the DeepCell models, we added a step to rescale the training images by a factor before using them in training. This factor was based on the image mpp that led to the best prediction accuracy from the previous section. For *tn_cyto* and *tn_nuclear*, the rescaling factors were 2/0.65 and 1.3/0.65, respectively, where 0.65 is the default *image_mpp* of DeepCell. For JACS, as in evaluation, scaling is effectively controlled by the patch size using in training and we selected 256×256 to correspond to the size of training images in image set BBBC038v1.

**Figure 3:**
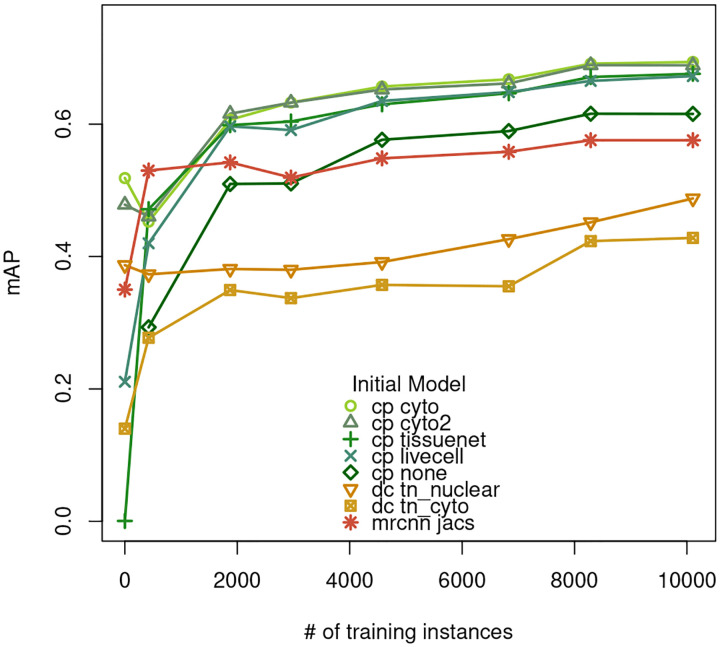
mAPs of the fine-tuned models. cp: Cellpose, dc: DeepCell. Each pre-trained model is trained seven times, each time adding a new training image in the order shown in Table 1. The x axis indicates the number of masks included in training.

We trained each of the eight selected models seven times, every time adding one more training image. The training images were ordered in the same sequence as which they were annotated and the prediction performance of the trained models was evaluated in the same way as in the previous section. Each Cellpose and Mask R-CNN model training was repeated three times with three random seeds and the evaluation results from the three runs are averaged. The mAPs are summarized in Figure 3 and Table 3. For the five Cellpose models, from 0 to 423 training instances (1 training image), *tissuenet* and *livecell* show a dramatic increase in performance, while the performance of *cyto* and *cyto2* actually drops. In other words, after the first training image, the performance of the four pre-trained Cellpose models converges. As the second training image is added, the number of training instances goes from 423 to 1873 and all five Cellpose models show a large improvement in performance. After two training images, performance still increases with additional data, but the rate of improvement slows to about *0.007 mAP per 1000 cells* (averaged across five models).

The two DeepCell models and the Mask R-CNN model exhibit similar trends as the Cellpose models. tn_nuclear performance drops as the first training image is added, *tn cyto* shows large improvements as the first two training images are added, jacs shows a large improvement as the first image is added. All three models show some continued improvement as more training images are added.

After trained with all 7 images or 10,000 training instances, *cp cyto* and *cp cyto2* perform the best, followed by *cp tissuenet* and cp livecell, which are better than cp none. All Cellpose models outperform the DeepCell and Mask R-CNN models.

To make a more complete comparison, we also computed the model performance in terms of Adjusted Rand Index (ARI) at a referee’s suggestion. ARI is a common measure used to compare the similarity between two data clusterings. The results of cell segmentation can be viewed as clusters of pixels, with each cluster corresponding to one cell. Thus, in the context of cell segmentation, the ARI between a set of predicted masks and the ground truth masks indicates how often a pair of pixels that should be in the same cell are actually segmented into the same cell, or how often a pair of pixels that should be in different cells are actually segmented into different cells. The ARI has a value between −1 and 1. A value of 0 indicates that the two predicted masks do not agree with the ground truth on any pair of points and are essentially random. A value of 1 indicates that the predicted masks are perfect. Though a value less than 0 is theoretically possible, it is unlikely to arise in real-world data. The last column in Table 3 shows that the fine-tuned Cellpose models have similar ARIs, ranging from 0.812 to 0.817, compared to 0.648 to 0.668 for the fine-tuned DeepCell models and 0.706 for the fine-tuned MR-CNN jacs model.

### Optimizing Cellpose performance

In the previous two sections we left most of the training and evaluation parameters at their default values. This is partly necessary because there are many models to compare and partly desirable because we wanted to compare different models as they were. The results from the previous section show that the Cellpose models initialized with the weights from cyto and trained with seven new training images perform the best in our test. Now we seek to optimize the training and evaluation of *cp cyto*-based models.

The first aspect of training we delve into is the size of the input to the convolutional neural network. By default, Cellpose takes a 224×224 patch from the center of the training image after random transformation and feeds it to its neural network. The choice of this patch size in a convolutional neural network goes back to at least [25]. To examine the effect of increasing or reducing the patch size, we compared its performance with three other choices of patch size. Each training was repeated three times with three random seeds and the evaluation results from the three runs were averaged. The results, summarized in Table 6, show that patch size has a large impact on performance and that the default choice 224×224 is much better than smaller patch sizes. Further increasing the patch size to 448×448 improves the mAP only by 0.004, but because there is little downside to choosing the largest patch size that is practical (patch size is limited by the amount of available GPU memory) and because the improvement is seen across all seven test images, we settled on patch size 448×448 for further experimentation. For inference, we keep the default patch size 224×224.

The second aspect of training we experimented with was the data augmentation step. Cellpose by default uses several data augmentation strategies, including random rotation, random flipping, random scaling, and random translation. Random translation is necessary for making use of the whole image when the patch size is different from the image size. To examine the effect of the other three transformations, cellpose package. Alternatively, general tools for augmentation in semantic segmentation and instance segmentation tasks can be used [26]. Each training was repeated three times with three random seeds and the evaluation results from the three runs were averaged. Note that at inference time no data augmentation was performed. The results, summarized in the right half of Table 6, show that turning off random rotation improves the mAP by 0.013, which is almost equivalent to adding 2000 training instances based on the estimate from the previous section (AP for individual images changes between −0.003 and 0.030). Turning off random flipping improves the mAP by 0.002 (AP for individual images changes between −0.007 and 0.013). Turning off random scaling decreases the mAP by 0.003 (AP for individual images changes between −0.023 and 0.018). We also tried turning off both random rotation and random flipping, which resulted in a change of −0.003 in mAP. It is worth noting that we used the same transformation for all seven training images and that for larger, more diverse training datasets, it is possible to apply different transformations to individual training images [27].

The results in Table 6 are the average mAP across replicates from three random seeds. To test whether this difference between turning random rotation on and off is statistically significant, we repeated the experiment three more times. The individual mAPs from the six replicates are shown in Supplemental Materials Table A.1. The Wilcoxon rank-based test comparing the mAPs with and without rotation returned a P-value of 0.031. The Wilcoxon rank-based tests comparing the mAPs with and without random scaling or random flipping both returned a P-value of 0.81. Based on these results, we recommend turning off random rotation in the data augmentation step in Cellpose training.

Thirdly, we experimented with collating four copies of each training image in a 2×2 formation and used it in training. Thus, each training image was measured 2318×2080 pixels. This did not lead to improvement in performance.

Lastly, we tested how batch size affected performance. Cellpose uses a default batch size of 8. We found that decreasing batch size led to worse performance.

Cellpose has two evaluation time parameters that control the post-processing of the neural network output into mask prediction. The first parameter is model fit threshold, which parameterizes a quality control step that is applied to the gradient flow output from the network. The default value is 0.4, and setting larger value returns more masks but some of them may be ill-shaped. The second parameter is *cellprob_threshold*, which is applied to the pixel probability map resulted from the network and only pixels above the threshold are considered in the post-processing step. The default is 0 on the logit scale, and setting larger value returns less masks. The results are summarized in Table 4 and show that the default values for these two thresholds selected by Cellpose perform the best, which lead to an mAP of 0.711 and an ARI of 0.826.

We further hypothesized that if we focused on masks above a certain size, the mAP would improve. We filtered both the ground truth masks and the predicted masks by the number of pixels in the masks and experimented with three thresholds:, 50, 100, and 200. The results are shown in (Table 5). A minimum mask size of 100 produced the best mAP 0.746.

To further understand the limiting factors on model performance, we asked the human expert to draw cells masks a second time for one of the test images, 2_CD3. Treating this set of masks as another set of predictions, we computed its AP against the first set of expert-drawn masks (without filtering by size) and got 0.662. An area where the two sets of expert-drawn masks differ is shown in Supplementary Figure A.5.

### Visualizing the predicted masks

**Figure 4:**
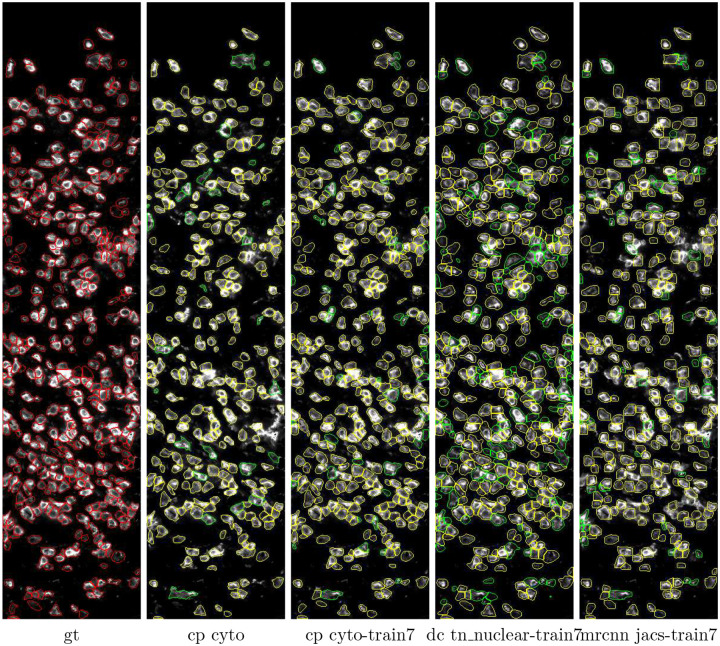
Predictions on a 2 CD3 test image. All panels show immunostaining with an anti-CD3 antibody. gt: ground truth, cp: Cellpose, dc: DeepCell, mrcnn: Mask R-CNN. Ground truth masks are shown in red. True positive and false positive predicted masks (IoU threshold 0.5) are shown in yellow and green, respectively.

To obtain a more intuitive understanding of how different models perform, we overlay the predicted marks from four models on top of the 2_CD3 test image in Figure 4. To facilitate comparison, we draw the predicted masks in two colors; the true positive masks are shown in yellow while the false positive masks are shown in green. The ground truth masks are shown in red as reference in the left-most panel. Comparing the three train7 models on the right, we see that the DeepCell model predictions have more green (false positive) masks than the other two, while the Mask R-CNN model predictions have fewer yellow (true positive) masks than the other two. Comparing the *cp cyto* and *cp cyto-train7* models, we see that the cp cyto-train7 panel has more yellow masks than the *cp cyto* panel.

**Figure 5:**
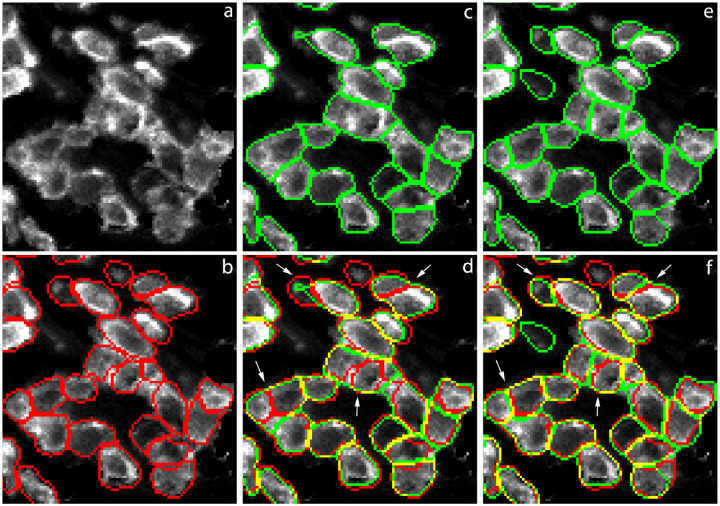
A magnified portion of the 2_CD3 test image near the lower-right corner. All panels show immunostaining with an anti-CD3 antibody. Ground truth masks are shown in red in panels b, d, and f. *cyto*-predicted masks are shown in green in panels c and d. *cyto-train7*-predicted masks are shown in green in panels e and f. The four white arrows indicate places where *cyto-train7* shows improvements upon *cyto*.

To get a more detailed look at the differences between *cp cyto* and *cp cyto-train7*, we zoom into an area near the lower-right corner of the 2_CD test image in Figure 5. The four white arrows in panel (d) and (f) point to places where *cyto-train7* correctly segments clusters of cells while cyto fails to. Another example of clustered cells is shown in Figure A.4 of the Supplementary Materials. From these images we see that when two cells stained with surface markers are very close to each other, the border between the two cells tends to be stained more brightly. This is one of the visual cues that human experts use in their work and *cp cyto-train7* appears to be able to mimic that to some extent.

## Conclusion

Accurate cell segmentation is key to all the downstream analyses in spatial molecular profiling. Due to the potential difficulty in distinguishing individual cells within densely packed tissues, segmentation based on cell surface markers allows for precise segmentation in a way that is not possible using only nuclear and cytoplasmic markers. In this paper we presented a set of annotated immunofluorescence images of cell surface markers stained HSV-infected skin. We investigated the performance of three types of deep learning models for cell segmentation both before and after training on this imageset. The best model after training*, cyto-train7*, achieved a superhuman performance and adds a valuable tool for precise segmentation of cells in dense tissues for the field.

**Figure 6:**
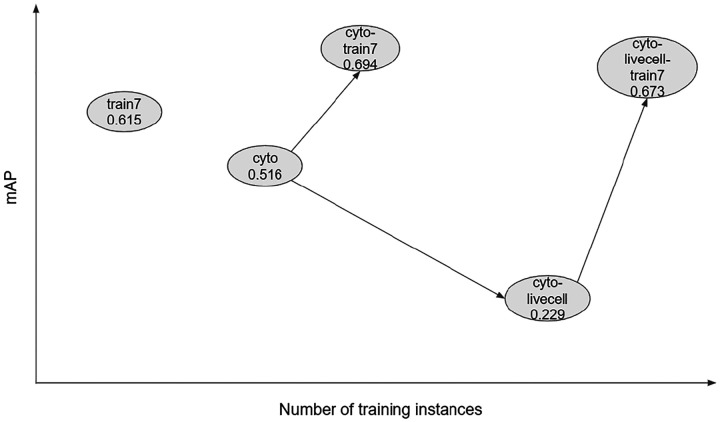
Relationships between four Cellpose models. *Cyto* is trained with ~70,000 cells from the Cellpose imageset [9]. Starting from *cyto*, *cyto-livecell* is obtained by training with ~80,000 cells from the LiveCell imageset [12] and *cyto-train7* is obtained by training with ~10,000 cells from the current imageset. *Cyto-livecell-train7* is obtained by starting from *cyto-livecell* and training with the current imageset. The mAPs for the test images from the current imageset are shown under each model.

We first evaluated eighteen deep learning-based cell segmentation models that are either pre-trained or trained by us using public imagesets, and found the mAP to range from 0.001 to 0.516. We fine-tuned eight of these models using more than 10,000 training instances from seven immunofluorescence images and found that prediction performance improved substantially after training. The best model after training was the one initialized with weights from the Cellpose cyto model, which increased the mAP from 0.516 (no fine tuning) to 0.694. The relationships between five different Cellpose models are shown schematically in Figure 6. Interestingly, training dramatically improved the performance of the two Cellpose models *cyto-tissuenet* and *cyto-livecell*, increasing the mAP from 0.001 to 0.676 and from 0.229 to 0.673, respectively, almost matching cyto-train7. One way of thinking about these results is that training with our imageset ”unlocks” capabilities that *cyto-tissuenet* and *cyto-livecell* already have, but are obscured in their respective additional training processes. These results highlight both the plasticity of the Cellpose models and the robust performance of the *cyto* model as a pre-trained model for further training using different imagesets.

We also trained a Cellpose model from randomly initialized weights. Figure 3 shows that as the number of training instances increases, the gap in performance between starting from cyto and starting from random weights remains relatively constant, even as the rates of gain for both curves slow down. This suggests that the training signal in our imageset alone is not sufficient and that initializing with *cyto*, which is trained with a diverse set of images from the Cellpose imageset, is essential.

We further optimized training of the Cellpose models and found that removing random rotation in the data augmentation step further increased mAP by 0.013 (P value 0.031 across replications with different random seeds). One potential explanation to this surprising result is that rotation is not a loss-free transformation and some signals may be distorted in the transformed images. This may not matter for classification tasks, where data augmentation techniques were originally developed, but for segmentation tasks, it could pose a problem. In fact, in medical image segmentation, that random rotation may hurt performance has been widely recognized [28–30, e.g.]. Consistent with this explanation is the observation that the flip-only transformation, which does not lead to quality loss, is also not associated with performance degradation (Table 6).

The mAP of the optimized Cellpose model in the current imageset is 0.711, which could ideally be further improved upon. More training data may improve the mAP as the results in Table 3 show that the average mAP of the five Cellpose models improves at a rate of 0.007 per 1000 cells from Train2 and Train7. However, returns diminish at some point when continuing to add more training data. For example, with *cyto* mAP improved by 0.026 from Train2 to Train3, but only by 0.003 from Train6 to Train7. The quality of training data may also play a significant role in prediction accuracy. This is supported by two lines of evidence. First, since a slice of tissue may go through a cell at a high latitude, the size of a cell in the image may include a range. It is hard to be categorical about the ground truth at the low end of the range. Thus, restricting to masks above a certain size threshold may result in higher accuracy. Our results supported this hypothesis. The mAP improved from 0.711 to 0.746 with minimum size 100 pixels (Table 5), which is equivalent to adding 5000 training masks based on the estimate of 0.007 mAP/1000 cells. Second, the *cyto-train7* model achieved an AP of 0.746 on the test portion of 2_CD3 (Table 4, column 1), which is higher than that achieved by the human expert (the masks drawn by the same human expert on a different day for this test image had an AP of 0.662 relative to the original ground truth masks). This suggests that even human experts may have difficulty making consistent calls, most likely due to limits on the signal-to-noise ratio in the imageset. We conjecture that better segmentation accuracy may come from training data of higher quality, e.g. increased image resolution.

## Figures and Tables

**Table 1:** Numbers of ground-truth masks generated in each of the seven images.

	Training	Testing	Training+Testing
1_CD8	423	127	550
2_CD3	1450	417	1867
3_CD4	1082	282	1364
4_CD3	1620	347	1967
5_CD3	2255	402	2657
6_CD3	1458	437	1895
7_CD3	1818	259	2077
Total	10106	2271	12377

**Table 2:** Average precision of the pre-trained models.

	1	2	3	4	5	6	7	mAP
	CD8	CD3	CD4	CD3	CD3	CD3	CD3	
Cellpose								
cyto	0.623	0.570	0.669	0.455	0.409	0.480	0.406	0.516
cyto2	0.561	0.534	0.584	0.437	0.407	0.462	0.403	0.484
nuclei	0.217	0.283	0.135	0.234	0.231	0.267	0.144	0.216
tissuenet	0.000	0.000	0.004	0.000	0.000	0.000	0.000	0.001
livecell	0.392	0.178	0.356	0.158	0.137	0.123	0.256	0.229
TN1	0.008	0.000	0.000	0.000	0.000	0.000	0.000	0.001
TN2	0.000	0.000	0.004	0.000	0.000	0.000	0.000	0.001
TN3	0.000	0.000	0.004	0.000	0.000	0.000	0.000	0.001
LC1	0.252	0.179	0.199	0.117	0.136	0.165	0.144	0.170
LC2	0.293	0.122	0.243	0.086	0.112	0.038	0.104	0.142
LC3	0.479	0.267	0.457	0.241	0.208	0.219	0.226	0.300
LC4	0.276	0.131	0.263	0.124	0.109	0.096	0.173	0.167
DeepCell								
cytoplasm	0.225	0.402	0.398	0.297	0.145	0.146	0.202	0.259
nuclear	0.312	0.373	0.486	0.297	0.170	0.113	0.172	0.275
tn_cyto	0.248	0.163	0.175	0.105	0.092	0.081	0.115	0.140
tn_nuclear	0.385	0.467	0.432	0.373	0.434	0.374	0.244	0.387
Mask R-CNN								
CellSeg	0.466	0.427	0.478	0.408	0.340	0.390	0.316	0.404
jacs	0.383	0.385	0.423	0.338	0.316	0.344	0.293	0.355

**Table 3:** Performance of the fine-tuned models. Each pre-trained model is trained seven times. From Train1 to Train7, new training images are added one at a time in the order shown in Table 1. For train7, two performance metrics are shown: mAP and ARI, where ARI is the average Adjusted Rand Index over seven test images.

	mAP								ARI
	Pretrained	Train1	Train2	Train3	Train4	Train5	Train6	Train7	Train7
Cellpose									
cyto	0.518	0.453	0.607	0.633	0.657	0.668	0.691	0.694	0.815
cyto2	0.478	0.460	0.616	0.632	0.652	0.661	0.689	0.689	0.817
tissuenet	0.001	0.471	0.598	0.604	0.630	0.647	0.671	0.676	0.813
livecell	0.211	0.420	0.597	0.591	0.635	0.648	0.665	0.673	0.812
none		0.293	0.510	0.510	0.576	0.589	0.616	0.615	0.791
DeepCell									
tn_nuclear	0.387	0.373	0.381	0.380	0.392	0.426	0.452	0.488	0.668
tn_cyto	0.140	0.277	0.349	0.337	0.357	0.355	0.423	0.428	0.648
MR-CNN									
jacs	0.350	0.530	0.542	0.519	0.548	0.558	0.576	0.576	0.706

**Table 4:** Effect of evaluation time parameters on the average precision of Cellpose models trained with seven training images, initialized with *cyto* weights, patch size 448×448, and no random rotation in the data augmentation step.

	default	flow threshold		prob threshold	
		0.3	0.5	−1	1
1_CD8	0.730	0.728	0.726	0.706	0.729
2_CD3	0.746	0.733	0.748	0.730	0.741
3_CD4	0.795	0.780	0.782	0.772	0.778
4_CD3	0.649	0.630	0.646	0.619	0.647
5_CD3	0.699	0.664	0.698	0.666	0.687
6_CD3	0.710	0.684	0.703	0.661	0.716
7_CD3	0.648	0.623	0.640	0.621	0.657
mAP	0.711	0.692	0.706	0.682	0.708

**Table 5:** Effect of minimum mask size on the average precision of Cellpose models trained with seven training images, initialized with *cyto* weights, patch size 448×448, and no random rotation in the data augmentation step.

	min size			
	0	50	100	200
1_CD8	0.730	0.735	0.710	0.697
2_CD3	0.746	0.750	0.774	0.669
3_CD4	0.795	0.800	0.822	0.753
4_CD3	0.649	0.663	0.731	0.637
5_CD3	0.699	0.701	0.724	0.646
6_CD3	0.710	0.712	0.727	0.615
7_CD3	0.648	0.649	0.736	0.586
mAP	0.711	0.716	0.746	0.658

**Table 6:** Effect of patch size and data augmentation on the average precision of Cellpose models trained with seven training images initialized with cyto weights.

	56×56	112×112	224×224	448×448	448×448		
	full data augmentation				no rotate	no flip	no scale
1_CD8	0.646	0.671	0.703	0.718	0.730	0.720	0.695
2_CD3	0.587	0.697	0.749	0.749	0.746	0.752	0.753
3_CD4	0.668	0.734	0.762	0.765	0.795	0.773	0.751
4_CD3	0.503	0.600	0.628	0.644	0.649	0.639	0.649
5_CD3	0.491	0.616	0.690	0.690	0.699	0.687	0.693
6_CD3	0.555	0.644	0.686	0.687	0.710	0.700	0.705
7_CD3	0.450	0.575	0.639	0.635	0.648	0.628	0.617
mAP	0.557	0.648	0.694	0.698	0.711	0.700	0.695

## Data Availability

The imageset is contained within the GitHub repository: https://github.com/youyifong/dense_cell_segmentation.
